# Effect of Vitamin D Supplementation on Cardiometabolic Risks and Health-Related Quality of Life among Urban Premenopausal Women in a Tropical Country – A Randomized Controlled Trial

**DOI:** 10.1371/journal.pone.0110476

**Published:** 2014-10-28

**Authors:** Mazliza Ramly, Moy Foong Ming, Karuthan Chinna, Suhaili Suboh, Rokiah Pendek

**Affiliations:** 1 Department of Social and Preventive Medicine, Faculty of Medicine, University of Malaya, Kuala Lumpur, Malaysia; 2 Julius Centre University of Malaya, Department of Social and Preventive Medicine, Faculty of Medicine, University of Malaya, Kuala Lumpur, Malaysia; 3 Department of Medicine, Faculty of Medicine, University of Malaya, Kuala Lumpur, Malaysia; University of Ottawa, Canada

## Abstract

**Background:**

Many observational studies linked vitamin D to cardiometabolic risks besides its pivotal role in musculoskeletal diseases, but evidence from trials is lacking and inconsistent.

**Aim:**

To determine whether Vitamin D supplementation in urban premenopausal women with vitamin D deficiency can improve cardiometabolic risks and health-related quality of life (HRQOL).

**Design:**

A double-blind randomized controlled trial was conducted in Kuala Lumpur, Malaysia. A total of 192 vitamin D deficient (<50 nmol/l) premenopausal women were randomized to receive either vitamin D 50,000 IU or placebo once a week for 2 months and then monthly for 10 months. Primary outcomes were serum 25(OH)D, serum lipid profiles, blood pressure and HOMA-IR measured at baseline, 6 months and 12 months. HRQOL was assessed with SF-36 at baseline and 12 months.

**Results:**

Ninety three and ninety-nine women were randomised into intervention and placebo groups respectively. After 12 months, there were significant differences in the serum 25(OH)D concentration (mean difference: 49.54; 95% CI: 43.94 to 55.14) nmol/l) and PTH levels (mean difference: −1.02; 95% CI: −1.67 to −0.38 pmol/l) in the intervention group compared to placebo group. There was significant difference between treatment group in both serum 25(OH)D and PTH. There was no effect of supplementation on HOMA-IR, serum lipid profiles and blood pressure (all p>0.05) between two groups. There was a small but significant improvement in HRQOL in the components of vitality (mean difference: 5.041; 95% CI: 0.709 to 9.374) and mental component score (mean difference: 2.951; 95% CI: 0.573 to 5.329) in the intervention group compared to placebo group.

**Conclusion:**

Large and less frequent dosage vitamin D supplementation was safe and effective in the achievement of vitamin D sufficiency. However, there was no improvement in measured cardiometabolic risk factors in premenopausal women. Conversely vitamin D supplementation improves some components of HRQOL.

**Trial Registration:**

Australian New Zealand Clinical Trial Registry ACTRN12612000452897

## Introduction

Vitamin D is well known for its fundamental role in bone mechanism and calcium homeostasis [1],[2]. More recently, many studies found that vitamin D may play an important role in the prevention of cardiovascular diseases and metabolic syndrome risk factors [2]–[6]. The mechanisms in which vitamin D may protect against these diseases include activation of the renin-angiotensin-aldosterone systems, increase arterial intimae thickness, enhancement in insulin secretion and insulin sensitivity and inflammatory cytokines [2]. Many observational studies have shown that vitamin D deficiency, defined as serum 25(OH)D<50 nmol/l or 20 ng/ml [7] is associated with cardiometabolic diseases [4],[8]–[10]. A meta-analysis of prospective studies by Khan et al [5] evaluated association between vitamin D levels and metabolic outcomes in healthy adults, demonstrated a significant inverse association of vitamin D status with metabolic syndrome. However, findings from this review were not able to establish causality, which required robust evidence from clinical trials. Clinical trials on the other hand produced inconsistent results [11]–[17]. These trials had marked study design variation in terms of samples size, study duration, participants’ characteristics, primary outcomes, intervention dosage and formulation. Furthermore, existing randomized controlled trials were generally short duration (less than one year), insufficient dosage or underpowered.

Vitamin D deficiency is established risk factors for osteoporosis, falls and fractures and all these may impair HRQOL. Vitamin D deficiency can occur to any individuals regardless of age or health condition. Yet, assessment of HRQOL in vitamin D deficiency was only performed in elderly population. These observational studies found vitamin D deficiency to be inversely associated with mental and physical HRQOL [18]–[24]. Furthermore, most studies were restricted to disease-specific populations such as the postmenopausal osteoporotic women [18],[19],[22],[23], elderly with heart failure [25] and elderly on dialysis [20]. Nevertheless, one observational study was found to examine the association between vitamin D and HRQOL among healthy premenopausal women [26] and another assessed the association between vitamin D and HRQOL among elderly in community setting [24]. Both studies observed inverse association between vitamin D and HRQOL. To our best knowledge, so far there are no other clinical trials evaluating the effect of vitamin D supplements on HRQOL in a healthy population.

Therefore, we designed this double-blind randomized placebo-controlled trial to determine whether vitamin D supplement can improve cardiometabolic risk factors such as blood pressure (BP), homeostasis model assessment insulin resistance (HOMA-IR), triglycerides (TG) and high-density lipoprotein cholesterol (HDL) as well as HRQOL in urban pre-menopausal women who were vitamin D deficient.

## Method

The protocol for this trial is reported in full details in the published protocol [27]. The CONSORT checklist and Table S1 and Table S2 are available as supporting information; see Checklist S1, Table S1 and Table S2.

**Figure 1:**
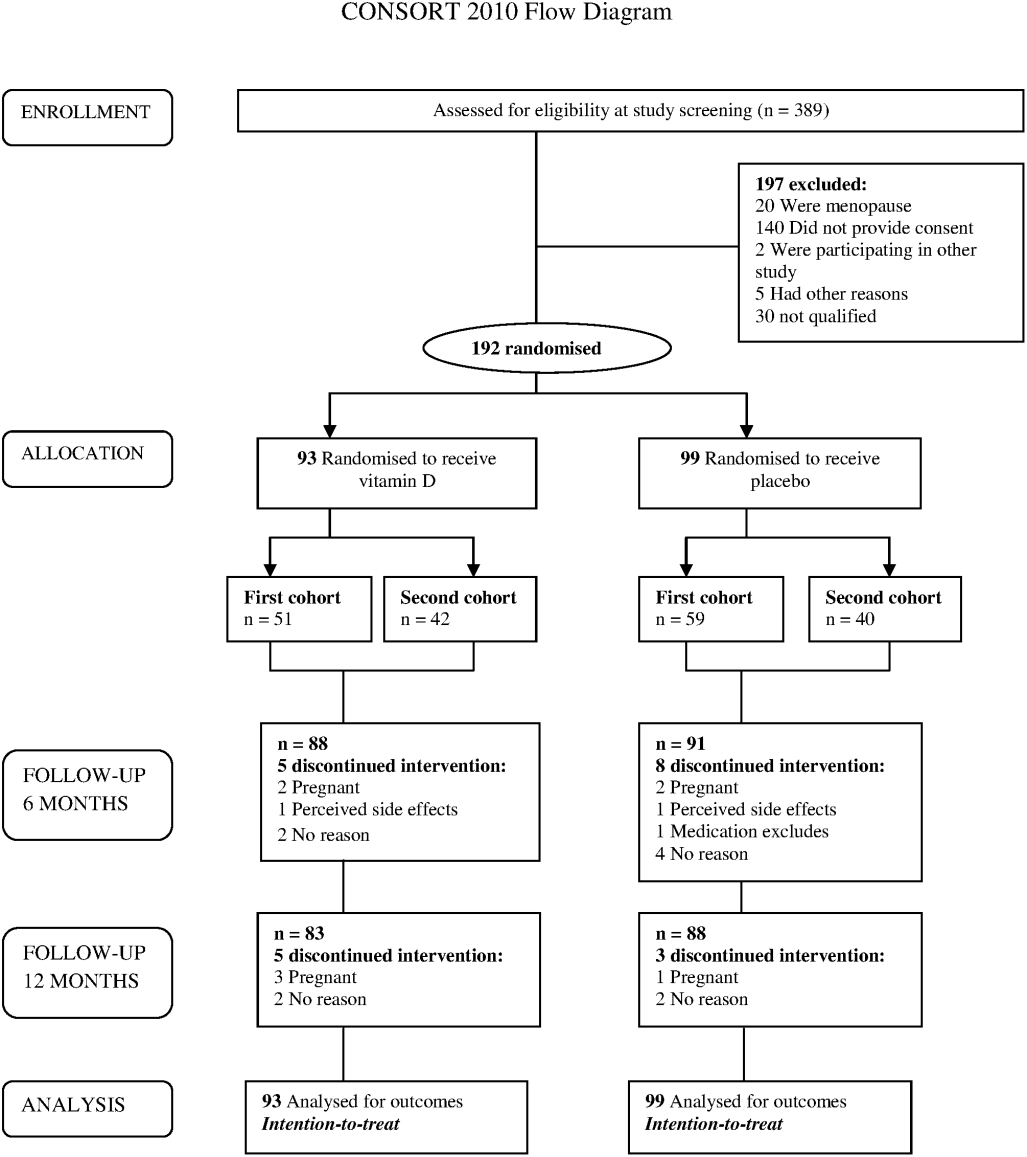
Flow diagram of the study, showing numbers of participants who were randomly assigned, received the intended treatment and were analyzed for the primary outcomes.

This was a randomized placebo-controlled, doubled-blind parallel trial. All premenopausal women aged 30 years old and above, working in a public university in Kuala Lumpur, Malaysia (*n = 389*) were screened for vitamin D deficiency (serum 25(OH)D≤20 ng/ml or ≤50 nmol/l). Exclusion criteria included abnormal serum PTH level (reference range for healthy adult was 1.1–7.3 pmol/l) and serum calcium (>2.7 nmol/l), known to have illnesses such as granuloma forming disorder, lymphoma, sarcoidosis or any types of cancer and taking daily vitamin D supplementation >1000 IU/day as well as pregnant women. Finally, 192 subjects were recruited in this trial (Figure 1).

The study was divided into two phases. Recruitment and screening including baseline clinical and anthropometry measurements were obtained in Phase one. All selected participants who meet the inclusion criteria in Phase one was ultimately invited to participate in this trial and proceed to Phase two. Written informed consent was obtained from these selected participants at this phase. The randomization and intervention of the first cohort (*n = 110*) commenced in October 2012 while the second cohort (*n = 82*) commenced in January 2013 (Phase two) (Figure 1). Intervention consisted of 50,000 IU (0.5 gram) of cholecalciferol (25(OH)D3) powder or placebo (0.5 gram) taken orally by diluting the powder into warm water before consumption. The consumption frequency was once a week for 8 weeks (equivalent to 7142 IU/day) and then once a month for 10 months (equivalent to 1667 IU/day) [7]. The consumption of 50,000 IU of vitamin D per week or per month is considered to be safe as the tolerable upper intake level for vitamin D is 10,000 IU per day [28]. We provided treatment for participants in the placebo group for 4 months after the trial completed, as it was unethical to withhold treatment. To ensure compliance with the intervention, participants were required to consume the active supplements or placebo in front of researcher during every follow-up. For those who did not attend the appointment, the researcher visited them at their workplace to give them the active supplements or placebo personally.

The randomization sequence was created using GraphPad Software with a 1∶1 allocation by a staff (AC) with no involvement in the trial. The participant’s names were matched with the random number sequence. The names of participants from intervention and placebo were printed and pasted on to the tubes filled with either vitamin D powder or placebo. The contents were not labelled on the bottles to maintain the allocation concealment. The tubes used were identical for both active intervention and placebo. Allocation sequence data was kept by AC in a secure place so that it could not be accessed or influenced by anyone, including the researchers. Outcomes measurements, randomization and allocation were fully concealed from the researchers, staffs involved in the trial and participants until the completion of data collection.

The primary outcome measures were serum 25(OH)D, systolic blood pressure, diastolic blood pressure, fasting blood insulin, fasting blood glucose, HOMA-IR, triglycerides, high-density lipoprotein cholesterol, low-density lipoprotein cholesterol and HRQOL using the SF-36 questionnaire.

Participants were recalled for clinical and anthropometry measurements at 6 months and 12 months. Serum calcium results at 6 months were checked for abnormality to detect any adverse reaction due to high dose of vitamin D supplementation. Participants were also advised to contact the researchers immediately if they suspected a reaction to supplements. Participants who were found to be pregnant at some point in the trial were also excluded in view of ethical consideration.

Methods for all measurements, questionnaires and laboratory analysis are reported in full details in the published protocol [27]. Homeostasis model assessment of insulin resistance (HOMA-IR) was used to establish a measure for insulin resistance. It can be calculated from a simple linear equation based on pairing FBS and Fasting Blood Insulin to establish a measure for insulin resistance: HOMA-IR = FBS(mmol/L)×Fasting Blood Insulin/22.5 [21]. Vitamin D concentration was analyzed using electrochemiluminescence immunoassay (ECLIA) vitamin D3 (25-OH) method on the Cobas E-411 analyzer. All tests and analyses were carried out by the Clinical Diagnostic Laboratory, University Malaya Medical Centre.

### Statistical Methods

A minimum of 88 subjects was required for each arm of the trial to demonstrate a significant difference at 80% power and a two-sided 5% significance. The calculations were based on the results by Zittermann et al. [29] which achieved a reduction in triglycerides of 0.19±0.54 mmol/L in vitamin D group while there is an increment of 0.03±0.50 in placebo group. Assuming a drop-out rate of 10%, 97 subjects would need to be recruited per arm. Sample size calculation was conducted using the OpenEpi Software version 2.3.1.

For baseline characteristics, descriptive statistics were used. Categorical data was described using count and percentages. All numerical data was checked for normality. Normally distributed variables were presented as mean ± standard deviation while abnormally distributed variables were presented as median ± interquartile range. Statistical comparisons were performed with independent *t-test*, Mann-Whitney U test or Chi-square test as appropriate. P-value was preset at 0.05. Some of the cases were loss to follow-up, therefore linear mixed effects procedure was used to test the differences in primary outcomes from baseline to follow-up at 6 months and 12 months. Data were analyzed according to the intention-to-treat principle. We also performed a linear mixed effects sensitivity analysis using complete cases. All statistical analysis was performed using the SPSS software version 16.0 (SPSS Inc, 2009, Chicago, Illinois).

### Ethical Approval

This trial was approved by the Medical Ethics Committee of University Malaya Medical Centre (reference number 907.22). Trial registration no: ACTRN12612000452897. Written informed consent was obtained from all participants.

## Results

Figure 1 shows the flow of participants in the trial. Three hundred eighty-nine potentially suitable participants were identified and invited for screening. Of these, a total of 192 women were randomly assigned to either intervention or placebo group. In total 21 women withdrew from the trial due to pregnancy, perceived side effects (amenorrhea) or no particular reasons. There were no reports of vitamin D intoxication or adverse reaction. A total of 171 participants completed the follow-up of 12 months.

### Baseline Data

Baseline characteristics of the participants are shown in Table 1. Both groups were similar in all the reported measures (P>0.05). Their mean age was 42±5 years, majority were Malays (>89%) and had at least secondary education (92%). A total of 15.6% of the participants had high cholesterol, 8.9% had hypertension and only 1% had diabetes mellitus. The mean scores for the HRQOL components were in the range of 60 to 70.

**Table 1:** Baseline socio-demographic characteristics of the intervention and placebo groups.

Characteristics	Intervention (n = 93)	Placebo (n = 99)	P-value^a^
	N (%)	N (%)	N (%)
**Ethnicity**			
Malay	83 (89.3%)	89 (89.9%)	0.777
Chinese	3 (3.2%)	3 (3.0%)	
Indian	7 (7.5%)	6 (6.1%)	
Others	0 (0%)	1 (1.0%)	
**Level of education**			
Primary	6 (6.5%)	8 (8.1%)	0.496
Secondary	43 (46.2%)	38 (38.4%)	
Tertiary	44 (47.3%)	53 (53.5%)	
**History of CVD**			
Diabetes mellitus	1 (1.1%)	1 (1.0%)	0.965
Hypertension	9 (9.7%)	8 (8.1%)	0.697
High cholesterol	15 (16.1%)	15 (15.2%)	0.852
Metabolic syndrome	26 (28%)	22 (22.2%)	0.359
	**Mean ± SD**	**Mean ± SD**	
Age (years)	42.58±5.35	42.88±4.99	0.69
BMI (kg/m^2^)	27.23±5.49	27.23±5.09	0.99
**Blood pressure**			
Systolic (mmHg)	121.61±16.19	118.90±14.85	0.23
Diastolic (mmHg)	77.77±10.64	76.80±10.99	0.53
Serum 25(OH)D (nmol/l)	30.19±8.71	29.99±9.70	0.88
Serum calcium (mmol/l)	2.18±0.07	2.19±0.08	0.246
Serum PTH (pmol/l)	4.60±2.19	4.76±2.05	0.611
**Lipid values (mmol/l)**			
Total cholesterol	5.17±0.99	5.28±0.83	0.425
HDL – Cholesterol	1.45±0.49	1.44±0.35	0.846
LDL – Cholesterol	3.26±0.76	3.34±0.71	0.457
Triglycerides	1.15±0.53	1.17±0.62	0.770
**HRQOL (SF-36)**			
Physical functioning	74.68±20.23	70.96±24.24	0.252
Role physical	74.35±22.98	72.27±24.40	0.544
Bodily pain	73.13±19.64	71.13±18.31	0.467
General health	66.77±16.87	66.12±17.13	0.791
Vitality	65.27±11.98	61.76±13.88	0.063
Social functioning	78.77±19.50	74.26±21.85	0.134
Role emotional	78.92±22.37	76.26±23.99	0.428
Mental health	73.12±12.11	71.06±16.62	0.331
PCS	49.57±6.78	48.80±7.41	0.453
MCS	50.09±6.69	48.65±9.17	0.218
	**Median; IQR**	**Median, IQR**	
**Insulin resistance**			
Serum glucose (mmol/l)	4.80; 0.65	4.80; 0.70	0.871
Serum insulin (mU/L)	9.30; 9.95	9.20; 8.80	0.874
HOMA-IR	2.00; 2.28	2.00; 2.00	0.908

HOMA-IR, homeostasis model assessment insulin resistance; CVD, cardiovascular disease; PTH, parathyroid hormone; 25(OH)D, 25-hydroxyvitamin D; IQR, Interquartile range; PCS, Physical component score; MCS, Mental component score; BMI was calculated as weight (kilogram) divided by height (m^2^).

a Determined with independent t-test or Mann-Whitney test for continuous variables as appropriate and with χ^2^ test for categorical data.

### Effects Of Vitamin D Supplements On Cardiometabolic Risk Factors

Primary outcomes for both groups at baseline, 6 months and 12 months are presented (Table 2). Mean serum 25(OH)D concentrations in the intervention group increased drastically in the first 6 months (mean difference: 53.72; 95% CI: 49.23 to 58.18 nmol/l) followed with a small increment after that (mean difference: 1.83; 95% CI: −0.94 to 4.61 nmol/l). There was also significant increase in the mean serum 25(OH)D in the placebo group after 6 months (mean difference: 7.17; 95% CI: 1.46 to 12.85 nmol/l), however the level was still in the deficient category (<50 nmol/l). A total of 91.6% of the intervention group achieved 25(OH)D more than 50 nmol/l (20 ng/ml) and 63.4% achieved a level of 75 nmol/l (30 ng/ml) or higher. In contrast, 13.1% of the placebo group had 25(OH)D above 50 nmol/l and only 1% had a value of 75 nmol/l or higher. Mean PTH concentration remained suppressed in the intervention group as the mean serum 25(OH)D increased, however the change in PTH was not statistically significant. In the placebo group, the mean PTH concentration increased from baseline to 6 months (mean difference: 1.04; 95% CI: 0.49 to 1.60 pmol/l) but declined slightly at 12 months (mean difference: −0.58, 95% CI: −1.12 to −0.05 pmol/l). There were significant differences in mean concentrations of total 25(OH)D and PTH between treatment groups after 12 months of treatment.

**Table 2:** Summary of the outcome measurements overtime (baseline, 6 months and 12 months) – Intention-to-treat analysis.

	Intervention (n = 93)	Placebo (n = 99)	Mean difference(95% CI) betweentreatment group^a^
	Mean (95% CI)	Mean (95% CI)	
**Se 25(OH)D (nmol/l)**			
Baseline	30.19 (28.56 to 31.82)	29.98 (27.20 to 32.78)	0.19 (–2.43 to 2.82
6 month	83.91 (79.84 to 87.97)	37.15 (34.39 to 39.90)	**46.92 (42.42 to 51.43)**
12 months	85.74 (81.25 to 90.22)	36.09 (33.63 to 38.55)	**49.54 (43.94 to 55.14)** *
**Se PTH (pmol/l)**			
Baseline	4.60 (4.18 to 5.03)	4.76 (4.34 to 5.18)	–0.16 (–0.76 to 0.45)
6 month	4.58 (4.05 to 5.10)	5.86 (5.34 to 6.38)	**–1.28 (–2.02 to −0.55)** *
12 months	4.19 (3.74 to 4.66)	5.22 (4.77 to 5.67)	**–1.02 (–1.67 to −0.38)** *
**Se calcium (mmol/l)**			
Baseline	2.18 (2.17 to 2.19)	2.19 (2.18 to 2.21)	–0.01 (–0.03 to 0.009)
6 month	2.23 (2.21 to 2.26)	2.24 (2.21 to 2.26)	–0.005 (–0.04 to 0.03)
12 months	2.24 (2.22 to 2.26)	2.22 (2.20 to 2.24)	0.02 (–0.01 to 0.05)
**BMI**			
Baseline	27.22 (26.14 to 28.31)	27.23 (26.18 to 28.28)	–0.005 (–1.511 to 1.500)
6 month	28.08 (26.96 to 29.19)	27.76 (26.67 to 28.86)	0.313 (–1.251 to 1.877)
12 months	27.63 (26.45 to 28.78)	27.84 (26.73 to 28.95)	–0.209 (–1.807 to 1.389)
**Systolic BP**			
Baseline	121.6 (118.4 to 124.8)	118.9 (115.8 to 121.9)	2.71 (–1.71 to 7.13)
6 month	126.3 (122.9 to 129.6)	123.9 (120.7 to 127.2)	2.38 (–2.27 to 7.04)
12 months	125.8 (122.6 to 128.9)	123.9 (120.8 to 126.9)	1.89 (–2.56 to 6.35)
**Diastolic BP**			
Baseline	77.77 (75.56 to 79.99)	76.79 (74.65 to 78.94)	0.976 (–2.107 to 4.059)
6 month	79.74 (77.38 to 82.10)	79.23 (76.97 to 81.55)	0.508 (–2.805 to 3.820)
12 months	77.52 (75.22 to 79.81)	76.76 (74.53 to 78.99)	0.757 (–2.441 to 3.954)
**Se Glucose (mmol/l)**			
Baseline	5.07 (4.88 to 5.25)	4.93 (4.75 to 5.12)	0.13 (–0.13 to 0.39)
6 month	5.14 (4.97 to 5.32)	5.07 (4.89 to 5.24)	0.07 (–0.17 to 0.32)
12 months	5.04 (4.83 to 5.26)	5.11 (4.90 to 5.32)	–0.07 (–0.37 to 0.23)
**Se insulin (mU/L**			
Baseline	13.81 (10.38 to 17.24)	11.07 (7.74 to 14.39)	2.74 (–2.04 to 7.51)
6 month	13.11 (11.18 to 15.04)	12.17 (10.27 to 14.06)	0.943 (–1.75 to 3.65)
12 months	13.93 (11.47 to 16.38)	12.74 (10.36 to 15.12)	1.19 (–2.23 to 4.61)
**HOMA-IR**			
Baseline	3.72 (2.25 to 5.19)	2.47 (1.04 to 3.91)	1.25 (–0.81 to 3.31)
6 month	3.12 (2.57 to 3.67)	2.84 (2.29 to 3.38)	0.28 (–0.49 to 1.05)
12 months	3.19 (2.61 to 3.78)	2.99 (2.42 to 3.56)	0.21 (–0.61 to 1.03)
**TG (mmol/l)**			
Baseline	1.15 (1.03 to 1.26)	1.17 (1.06 to 1.29)	–0.03 (–0.19 to 0.14)
6 month	1.38 (1.25 to 1.51)	1.19 (1.07 to 1.32)	**0.19 (0.01 to 0.37)** *
12 months	1.36 (1.23 to 1.49)	1.22 (1.09 to 1.35)	0.14 (–0.33 to 0.33)
**HDL-C (mmol/l)**			
Baseline	1.45 (1.36 to 1.54)	1.44 (1.35 to 1.52)	0.01 (–0.11 to 0.13)
6 month	1.43 (1.36 to 1.49)	1.50 (1.44 to 1.57)	–0.08 (–0.18 to 0.02)
12 months	1.52 (1.44 to 1.59)	1.53 1.50 (1.43 to 1.58)	0.02 (–0.09 to 0.13)
**LDL-C (mmol/l)**			
Baseline	3.26 (3.11 to 3.41)	3.34 (3.19 to 3.48)	–0.08 (–0.29 to 0.13)
6 month	3.25 (3.09 to 3.39)	3.31 (3.17 to 3.46)	–0.07 (–0.28 to 0.15)
12 months	3.62 (3.44 to 3.79)	3.63 (3.46 to 3.80)	–0.01 (–0.26 to 0.23)

*Significant at p<0.05.

a Determined with linear mixed effect.

BMI did not declined in the intervention group between baseline to 6 months (mean difference: 0.85, 95% CI: −1.07 to 2.77 kg/m^2^), however these changes were not clinically significant and did not differ between treatment groups. There were significant differences in the change in serum calcium, systolic blood pressure and LDL within the intervention group (p<0.05). Serum calcium and LDL were significantly different over time within the placebo group (p<0.05). However, these changes did not differ between treatment groups and were not clinically significant. An increase in serum 25(OH)D in the intervention group induced a reduction in HOMA-IR whereas the reverse was observed in the placebo group. However, these changes were not statistically significant between treatment groups. Sensitivity analysis using complete cases showed similar results (Table S1).

There was a reduction in proportion of metabolic syndrome from 28% to 25.8% in the intervention group, while in placebo group, the proportion increased from 22.2% to 23.2%. There was no difference between groups (p = 0.762). Among participants with metabolic risks at baseline (n = 26 for intervention group and n = 22 for placebo group), there was a small but significant improvement (p = 0.021) in the proportion of low HDL in the intervention group at the end of the trial. Similar improvement was also noted in the proportions of high blood pressure, abdominal obesity and elevated glucose, however all these were not statistically significant (data not shown).

### Effects Of Vitamin D Supplements On Health-Related Quality Of Life

The health-related quality of life (HRQOL) of the intervention and placebo groups is presented in Table 3. At baseline, the mean scores of HRQOL components in the SF-36 were in between 60 to 70. Vitamin D supplement improved general health of the intervention group (mean difference: 3.65; 95% CI: 0.57 to 7.73). However, there was no significant difference when compared to the placebo group. Apparently there was a small but significant improvement in vitality (mean difference: 5.041; 95% CI: 0.709 to 9.374) and mental component score (mean difference: 2.951; 95% CI: 0.573 to 5.329) in the intervention group compared to placebo group. Sensitivity analysis using complete cases were also performed for HRQOL and it also showed similar results (Table S2).

**Table 3:** Summary of the health-related quality of life parameters overtime (baseline and 12 months) – Intention-to-treat analysis.

	Intervention (n = 93)	Placebo (n = 99)	Mean difference(95% CI) betweentreatment group^a^
	Mean (95% CI)	Mean (95% CI)	
**Physical functioning**			
Baseline	74.68 (70.81 to 78.84)	70.96 (66.13 to 75.79)	3.718 (–2.624 to 10.06)
12 months	71.81 (66.53 to 77.09)	71.48 (66.62 to 76.33)	0.330 (–6.792 to 7.452)
**Role physical**			
Baseline	74.36 (69.62 to 79.09)	72.27 (67.41 to 77.14)	2.082 (–4.662 to 8.826)
12 months	73.45 (67.94 to 78.95)	72.84 (67.78 to 77.90)	0.605 (–6.820–8.029)
**Bodily pain**			
Baseline	73.13 (69.09 to 77.17)	71.13 (67.48 to 74.78)	1.998 (–3.416 to 7.411)
12 months	72.93 (67.76 to 77.09)	69.31 (65.64 to 72.97)	3.621 (–1.891 to 9.132)
**General health**			
Baseline	66.77 (63.29 to 70.25)	66.12 (62.71 to 69.54)	0.653 (–4.188 to 5.494)
12 months	70.42 (66.85 to 73.99)	68.66 (65.57 to 71.75)	1.763 (–2.924 to 6.449)
**Vitality**			
Baseline	65.27 (62.80 to 67.74)	61.76 (58.99 to 64.53)	3.511 (–0.173 to 7.196)
12 months	65.53 (62.45 to 68.61)	60.49 (57.39 to 63.58)	**5.041 (0.709 to 9.374)** *
**Social functioning**			
Baseline	78.77 (74.76 to 82.79)	74.26 (69.91 to 78.62)	4.512 (–1.377 to 10.401)
12 months	80.27 (76.24 to 84.29)	75.11 (70.66 to 79.56)	5.151 (–0.804 to 11.107)
**Role emotional**			
Baseline	78.93 (74.32 to 85.53)	76.26 (71.48 to 79.62)	2.662 (–3.938 to 9.262)
12 months	80.52 (75.45 to 85.58)	74.52 (69.43 to 79.62)	5.995 (–1.138 to 13.129)
**Mental health**			
Baseline	73.12 (70.63 to 75.61)	71.06 (67.74 to 74.37)	2.058 (–2.065 to 6.180)
12 months	75.12 (72.16 to 78.08)	71.53 (68.25 to 74.82)	3.586 (–0.806 to 7.979)
**Physical component** **score**			
Baseline	49.57 (48.17 to 50.97)	48.80 (47.32 to 50.28)	0.772 (–1.249 to 2.793)
12 months	48.99 (47.51 to 50.47)	49.06 (47.79 to 50.32)	–0.069 (–1.997 to 1.860)
**Mental component** **score**			
Baseline	50.09 (48.71 to 51.46)	48.65 (46.82 to 50.48)	1.440 (–0.835 to 3.715)
12 months	51.34 (49.81 to 52.86)	48.39 (46.54 to 50.23)	**2.951 (0.573 to 5.329)** *

*Significant at p<0.05.

a Determined with linear mixed effect.

## Discussion

Our participants were urban, premenopausal women working in a public university in the capital city of Malaysia. Although they were vitamin D deficient, the proportion of them having non-communicable diseases (NCD) such as hypertension and diabetes was relatively low compared to the general population [30]. However, more than 20% of them had metabolic syndrome. Their mean BMI was in the overweight category. This reflected that our participants were at risks for CVD in the future. As expected, the participants’ scores for some of the HRQOL components, namely the physical functioning, role physical and social functioning were found to be lower than the general population [31].

In the present study, we demonstrated 12 months of vitamin D supplementation at a dosage of 50,000 IU per week for the first 2 months and 50,000 IU per month for the next 10 months increased serum 25(OH)D to sufficient level among our participants. However, correction of vitamin D deficiency did not improve HOMA-IR, HDL, LDL or triglycerides. Nevertheless, among participants with metabolic syndrome, vitamin D supplementation improved HDL level, offering a promising non-pharmacologic intervention in the prevention of metabolic syndrome. We also found that vitamin D supplements improved the HRQOL among the participants particularly in vitality and mental component scores.

Our results also showed the dosage and frequency of Vitamin D supplementation was safe and effective. More than 80% of participants from the intervention group achieved serum 25(OH)D above 50 nmol/l compared to only 13% from placebo group. These results were comparable with other studies which used daily dosage of vitamin D3 [16],[17]. Although all women in the intervention group received the same dosage of vitamin D, a small proportion did not achieve a sufficient level of circulating vitamin D. This could be due to heterogeneity among women such as obesity. On further exploration of data, 6 out of 7 of these women were obese (>25 kg/m^2^). This is probably because of a decreased in bioavailability of vitamin D that is sequestered in the fat of individuals with excess adipose tissue. Monthly dosage had advantage in terms of compliance. Von Hurst et al [16] reported that their participants found it difficult to comply with daily dosage of vitamin D. None of our participants reported any adverse effects of vitamin D clinically or biochemically.

Evidences from epidemiologic studies showed vitamin D deficiency was linked with cardiometabolic risks [9],[10],[32]–[40]. Vitamin D insufficiency induced alterations in the calcium flux of pancreatic b-cells thus reduced insulin secretion [41],[42] and this might influence on insulin resistance (IR). In addition, vitamin D insufficiency also induced elevations in PTH, which could adversely affect glucose metabolism. As expected, PTH levels decreased within the intervention group. On the other hand, the PTH levels also decreased within the placebo group. This could possibly due to unintentional increased consumption of dietary calcium in the placebo group because of Hawthorne effect. This could be the reason for no significant differences in insulin resistance or glucose levels between groups.

They were many clinical trials trying to establish the role of vitamin D deficiency in the pathogenesis of type 2 diabetes mellitus [11],[12],[14]–[17],[33],[34],[43]. However, the results had been inconsistent. Most of these trials failed to detect any improvement in the glucose levels and insulin resistance. Our findings were consistent with results of many other published studies, in which the vitamin D supplements appeared to have no effect on fasting glucose and HOMA-IR [11],[12],[14],[15],[17]. Reasons for these negative results could be due to the studies were short duration, had small sample size and different dosage used in vitamin D formulations. On the contrary, data from two clinical trials showed that vitamin D supplementation improved plasma glucose and insulin resistance [16],[43].

Systematic reviews and observational studies found associations between vitamin D deficiency and triglycerides, HDL and LDL [44]–[47]. However, results from trials had been inconsistent. Clinical trials by Brevlasky et al [11] and Wood et al [17] found an absence of improvement in lipid profiles, similar with our findings. On the other hand, two other clinical trials [13],[29] found significant decrease in lipid in the treatment group. Major et al [13] reported that total cholesterol:HDL and LDL:HDL ratio in the treatment group were significantly decreased. Whereas, in study by Zitterman et al [29] the serum triglycerides and LDL (p<0.001) were reduced after one year of treatment with 3320 IU of vitamin D daily among healthy overweight individuals with inadequate vitamin D levels. These inconsistent results could be due to variations in sample size, dosage and formulation of vitamin D supplement, characteristics of participants and duration of trials.

Our participants were generally healthy premenopausal women with vitamin D deficiency. Although 26% of them had history of CVD, majority had normal glucose and lipid values, which may be difficult to improve further with vitamin D supplement. However, individuals with higher baseline metabolic risks may benefit from vitamin D supplementation. Analysis on a subset of our participants with metabolic risks at baseline demonstrated an improvement in HDL among the intervention group. However, the null effect of vitamin D supplementation on other metabolic risk factors may be due to the small sample size of our participants with metabolic risks. Therefore, there might be inadequate power to detect the effectiveness of vitamin D on these components. Future studies should recruit women with vitamin D deficiency and at risks for metabolic syndrome or CVD.

The increase levels in some of the clinical indicators (systolic blood pressure, triglycerides, LDL-cholesterol) within both intervention and placebo groups was probably due to the advancing age that affected the pathogenesis of these indicators [48]. Twelve months of treatment with vitamin D may not be sufficient to produce a significant reduction in the cardiometabolic risk factors as more time may be needed for the vitamin D to correct the metabolic risks back to normal.

The optimal dose for vitamin D supplementation on cardiometabolic risk could also influence our results. Expert opinions for daily vitamin D dosage ranged from 600 to 2000 IU for prevention of cardiovascular or metabolic risk [28],[49],[50]. The Institute of Medicine Report [28] concluded that due to lack of evidence, they could not make any recommendation for non-skeletal outcomes. The method of vitamin D supplementation in our study (50,000 IU weekly for 2 months and once a month after that) differed from the daily doses (400–4000 IU) used in majority of other studies [12],[15]–[17],[43],[51]. We used the recommendation by Holick [7] which stated that for an effective vitamin D deficiency treatment, higher dosage was needed at initial stage to correct the vitamin D deficient status. However, this recommendation only corrected the vitamin D deficiency. There is no current recommendation on the optimum dosage needed for improvement of cardiometabolic outcomes. There are few other studies using similar dosage [52] or larger dose of vitamin D supplementation [14],[25],[53] but in shorter duration (less than 6 months). Although we did not find any effect of vitamin D supplementation on cardiometabolic risks, we cannot exclude the possibility that higher dosage of supplementation for a longer duration might help in the prevention of cardiometabolic risks among individuals with existing co-morbidities or cardiometabolic events such as myocardial infarct or stroke. However, a review article has indicate that negative outcomes could be predicted due to failure to maintain circulating vitamin D over time as with monthly, quarterly or yearly bolus vitamin D dosing [54]. It appears that for the optimal benefits of vitamin D supplementation, sufficient amount of vitamin D should be provided on a daily basis to ensure that stable circulating concentrations are maintained over time as vitamin D has short circulating half-life of vitamin D. These particular reasons might explain the varying results of clinical trials. Therefore, a large-scale prospective clinical trial is needed to examine large dosage of daily versus weekly and monthly vitamin D with the same primary outcome measures.

On the other hand, we found vitamin D supplements improved the HRQOL of participants particularly in vitality and mental component score. These improvements were statistically significant but clinically insignificant. However, it has some clinical relevance as vitality assessed energy and fatigue; and women with vitamin D deficiency usually feel tired, low energy and worn out. Vitamin D is a nuclear steroid hormone which thought to be involved in brain health and function as well as neuromuscular functions. Vitamin D receptors in the cell’s nucleus regulate the expression of target genes when bound to 1,25(OH)D. These receptors are expressed in areas of the brain important to behavioural regulation [55]. An observational study by Ecemis et al [26] found physical component score, mental component score, physical functioning score and vitality score were impaired in vitamin D deficient and insufficient healthy premenopausal women. Other studies did relate vitamin D status with depression [56]–[59] and mental component score [19]. Another observational study also concluded that women who were on daily vitamin D supplementation had higher mental HRQOL scores [19].

At baseline, our study showed slightly higher scores in bodily pain components compared to the general population [20]. Although muscle and bone pain was a common symptom of vitamin D deficiency, we did not find any significant improvement for physical components score. This may be due to non-specific definition of pain was used in the SF-36 questionnaire which we administered. For example, Huang et al [21] used VR-36 (Veteran Rand 36 items) questionnaire as well as VAS (visual analog scale) to evaluate bodily pain and found that vitamin D supplementation was effective to alleviate pain and improve other components of HRQOL. Similarly, Sakalli et al [60] used VAS and SF-36 questionnaire to detect the level of pain and they reported that single megadose of vitamin D was effective to increase QOL particularly physical functioning, role physical, bodily pain, general health, social functioning and also decrease non-specific musculoskeletal pain. On the contrary, another study that used a disease-specific tool (Minnesota Living with Heart Failure questionnaire) did not find any improvement in physical function or HRQOL in older patients with heart failure [25].

To date, very few studies examined the relationship of vitamin D and HRQOL among healthy premenopausal women without osteoporosis as the study population. So far, we only found study by Ecemis et al [26] on Vitamin D and HRQOL among the healthy premenopausal women. They reported significant association between vitamin D and HRQOL.

It is difficult to recommend sunlight exposure to improve vitamin D levels due to interpersonal variations in sunlight exposure as well as the risks of skin cancer due to excessive UV radiation. However, skin cancer is a rare disease in Asia as compared to Caucasians [61]. A study by Nurbazlin et al. [62] has found that urban women in Malaysia had significantly lower sunlight exposure and vitamin D status compared to rural women. This study shown that sun index were the major factors influencing vitamin D status in Malaysian women. A trial study on the effect of nutrition education and sun exposure on vitamin D status among postmenopausal Malay women also found serum vitamin D level was increased through nutrition education and sunlight exposure [63]. Therefore it is sensible to recommend additional sunlight exposure for Malaysian women especially those living in the urban. Besides that, the best way of preventing vitamin D deficiency is by food fortification. Food fortification has the dual advantage of being able to deliver nutrients to large segments of the population without requiring radical changes in food consumption patterns. A recent review study found vitamin D fortification using vegetable oil can be the efficacious way to increase intake of daily vitamin D in Southeast Asian countries [64]. In addition to fortification, supplementation can be the alternative solution with high and less frequent doses are advisable especially to high risk population such as elderly. Nevertheless, we need to learn more about the long term safety and efficacy of the high dosage, less frequent vitamin D supplementation.

## Limitations And Strength

Although all of our participants were vitamin D deficient, only small proportion of them was at risk for CVD as our study was conducted in a non-clinical setting. From the National Health and Morbidity Survey IV (NHMS IV) conducted in 2011, only 14.5% of women age more than 18 years old were diabetic while 31.6% were hypertensive [65]. Therefore, we had difficulty recruiting women with vitamin D deficiency as those with CVD risk. So, our study was not powered to detect the effect of vitamin D supplementation on cardiometabolic risks. We were also not able to examine the incidence of cardiovascular events due to the short duration. A larger sample size of high risk population for CVD with longer follow-up and higher intakes of vitamin D supplementation may be needed to establish whether steady normalization of vitamin D levels will translate to decrease in cardiovascular event incidence. An article by Heaney [66] has indicated that inadequate intakes of vitamin D may produce more than one disease by more than one mechanisms and may require several years for the consequent morbidity to be sufficiently evident to be clinically recognizable as “disease”. Similarly more established evident are needed to prove that higher intakes of vitamin D are required for its prevention. For example, preventing the osteoporosis requires almost 4 times the amount of vitamin D needed to prevent rickets and this knowledge takes years to be established.

Nevertheless, our study has several strengths that worth mention. One of them is its high quality of randomisation where there was concealment of allocation, double-blinding among the participant and researchers and placebo controlled design. This reduced bias in term of selection and information bias as self-reported HRQOL was subjective. Larger but less frequent dosage of vitamin D may be advantageous from a compliance perspective compared to daily doses. There was also no problem with seasonal confounders as Malaysia is blessed with sufficient sunshine all year round necessary for cutaneous synthesis of vitamin D. Both groups were comparable in outdoor physical activity, sunlight exposure activity and diet at baseline and there were no changes pre and post intervention. In addition, our participants’ retention rate was good with only 11% drop-out from the study.

## Conclusion

This trial showed that Vitamin D supplementation over one year to premenopausal vitamin D deficient women living in an urban tropical country had no meaningful effect on measured cardiometabolic risk factors, but it did improve some components of HRQOL. Our findings provided evidence for the larger dosage but lower frequency to be effective in improving serum vitamin D levels. Further long term trials of this intervention versus daily and weekly vitamin D dosing especially among the high risk individuals with existing co-morbidities are needed.

## Supporting Information

Table S1 **Sensitivity analysis using complete cases of the outcome measurements overtime (baseline, 6 months and 12 months).**
(DOCX)

Table S2 **Sensitivity analysis using complete cases of the health-related quality of life parameters overtime (baseline and 12 months).**
(DOCX)

Checklist S1 **CONSORT Checklist.** CONSORT 2010 checklist of information to include when reporting a randomized trial. (DOC)
