# Supplementary material for: Effect of Vitamin D Supplementation on Cardiometabolic Risks and Health-Related Quality of Life among Urban Premenopausal Women in a Tropical Country – A Randomized Controlled Trial
Source: PLoS One. 2014 Oct 28;9(10):e110476. doi: 10.1371/journal.pone.0110476 (PMC4211685; doi:10.1371/journal.pone.0110476)
Supplement: Table S1 — (DOCX) [file pone.0110476.s001.docx]

Table S1: Sensitivity analysis using complete cases of the outcome measurements overtime (baseline, 6 months and 12 months)

|  | Intervention (n = 93) | Placebo (n = 99) | Mean difference (95% CI) between treatment group^a^ |
| --- | --- | --- | --- |
|  | Mean (95% CI) | Mean (95% CI) |  |
| Se 25(OH)D (nmol/l) |  |  |  |
| Baseline | 30.43 (28.54 to 32.32) | 30.17 (28.13 to 32.22) | 0.26 (-2.51 to 3.02) |
| 6 month | 83.88 (79.46 to 88.30) | 37.41 (34.89 to 39.94) | **46.47 (41.40 to 51.53)*** |
| 12 months | 85.77 (80.81 to 90.72) | 36.23 (33.57 to 38.88) | **49.54 (43.94 to 55.14)*** |
| Se PTH (pmol/l) |  |  |  |
| Baseline | 4.51 (4.03 to 4.98) | 4.76 (4.36 to 5.15) | -0.25 (-0.86 to 0.36) |
| 6 month | 4.49 (4.04 to 4.96) | 5.81 (5.27 to 6.34) | **-1.31 (-2.01 to -0.61)*** |
| 12 months | 4.19 (3.69 to 4.71) | 5.22 (4.81 to 5.63) | **-1.02 (-1.67 to -0.37)*** |
| Se calcium (mmol/l) |  |  |  |
| Baseline | 2.19 (2.17 to 2.20) | 2.19 (2.17 to 2.20) | -0.002 (-0.023 to 0.020) |
| 6 month | 2.23 (2.20 to 2.26) | 2.24 (2.22 to 2.25) | -0.004 (-0.038 to 0.031) |
| 12 months | 2.24 (2.22 to 2.26) | 2.22 (2.20 to 2.24) | 0.017 ( (-0.011 to 0.046) |
| BMI |  |  |  |
| Baseline | 27.47 (26.34 to 28.58) | 27.54 (26.47 to 28.61) | -0.066 (-1.598 to 1.466) |
| 6 month | 27.92 (26.79 to 29.06) | 27.85 (26.75 to 28.94) | 0.079 (-1.488 to 1.645) |
| 12 months | 27.63 (26.52 to 28.74) | 27.84 (26.68 to 28.99) | -0.209 (-1.804 to 1.386) |
| Systolic BP |  |  |  |
| Baseline | 122.3 (118.9 to 125.8) | 119.3 (116.2 to 122.4) | 3.07 (-1.53 to 7.66) |
| 6 month | 126.7 (123.0 to 130.5) | 123.9 (120.9 to 127.0) | 2.79 (-1.98 to 7.57) |
| 12 months | 125.8 (122.6 to 128.9) | 123.9 (120.7 to 127.0) | 1.89 (-2.55 to 6.34) |
| Diastolic BP |  |  |  |
| Baseline | 78.07 (75.83 to 80.31) | 76.88 (74.58 to 78.17) | 1.197 (-1.987 to 4.382) |
| 6 month | 79.83 (77.34 to 82.33) | 79.27 (77.01 to 81.54) | 0.559 (-2.788 to 3.905) |
| 12 months | 77.52 (75.15 to 79.89) | 76.76 (74.57 to 78.95) | 0.757 (-2.446 to 3.959) |
| Se Glucose (mmol/l) |  |  |  |
| Baseline | 5.08 (4.82 to 5.35) | 4.96 (4.81 to 5.10) | 0.13 (-0.17 to 0.43) |
| 6 month | 5.13 (4.91 to 5.36) | 5.08 (4.84 to 5.22) | 0.05 (-0.21 to 0.31) |
| 12 months | 5.04 (4.81 to 5.27) | 5.11 (4.91 to 5.31) | -0.07 (-0.37 to 0.23) |
| Se insulin (mU/L |  |  |  |
| Baseline | 14.28 (9.05 to 19.51) | 11.29 (9.66 to 12.93) | 2.99 (-2.48 to 8.45) |
| 6 month | 12.92 (11.07 to 14.77) | 12.19 (10.19 to 14.21) | 0.72 (-1.99 to 3.44) |
| 12 months | 13.93 (11.30 to 16.55) | 12.74 (10.49 to 14.99) | 1.19 (-2.25 to 4.62) |
| HOMA-IR |  |  |  |
| Baseline | 3.90 (2.01 to 5.79) | 2.53 (2.43 to 2.66) | 1.37 (-0.79 to 3.53 |
| 6 month | 3.06 (2.51 to 3.61) | 2.84 (2.30 to 3.41) | 0.21 (-0.57 to 0.98) |
| 12 months | 3.19 (2.61 to 3.78) | 2.99 (2.41 to 3.56) | 0.21 (-0.61 to 1.03) |
| TG (mmol/l) |  |  |  |
| Baseline | 1.20 (1.08 to 1.31) | 1.19 (1.05 to 1.32) | 0.012 (-0.162 to 0.187) |
| 6 month | 1.39 (1.24 to 1.55 | 1.20 (1.09 to 1.31) | **0.192 (0.006 to 0.378)*** |
| 12 months | 1.36 (1.21 to 1.51) | 1.22 (1.10 to 1.34) | 0.14 (-0.049 to 0.329) |
| HDL-C (mmol/l) |  |  |  |
| Baseline | 1.45 (1.34 to 1.56 | 1.45 (1.38 to 1.53) | 0.00 (-0.14 to 0.13) |
| 6 month | 1.44 (1.37 to 1.52) | 1.50 (1.44 to 1.57) | -0.06 (-0.16 to 0.04) |
| 12 months | 1.52 (1.44 to 1.60) | 1.50 (1.43 to 1.57) | 0.02 (-0.09 to 0.13) |
| LDL-C (mmol/l) |  |  |  |
| Baseline | 3.29 (3.13 to 3.47) | 3.29 (3.15 to 3.45) | -0.001 (-0.226 to 0.223) |
| 6 month | 3.25 (3.09 to 3.42) | 3.31 (3.17 to 3.46) | -0.059 (-0.277 to 0.160) |
| 12 months | 3.62 (3.42 to 3.81) | 3.63 (3.47 to 3.78) | -0.013 (-0.261 to 0.236) |

* Significant at p < 0.05

^a^ Determined with linear mixed effect
