# Supplementary material for: Effect of Vitamin D Supplementation on Cardiometabolic Risks and Health-Related Quality of Life among Urban Premenopausal Women in a Tropical Country – A Randomized Controlled Trial
Source: PLoS One. 2014 Oct 28;9(10):e110476. doi: 10.1371/journal.pone.0110476 (PMC4211685; doi:10.1371/journal.pone.0110476)
Supplement: Table S2 — (DOCX) [file pone.0110476.s002.docx]

Table S2: Sensitivity analysis using complete cases of the health-related quality of life parameters overtime (baseline and 12 months)

|  | Intervention (n = 93) | Placebo (n = 99) | Mean difference (95% CI) between treatment group^a^ |
| --- | --- | --- | --- |
|  | Mean (95% CI) | Mean (95% CI) |  |
| Physical functioning |  |  |  |
| Baseline | 74.28 (60.81 to 78.75) | 71.14 (65.96 to 76.32) | 3.14 (-3.65 to 9.94) |
| 12 months | 71.81 (66.53 to 77.09) | 71.48 (66.62 to 76.33) | 0.33 (-6.79 to 7.45) |
| Role physical |  |  |  |
| Baseline | 73.75 (68.79 to 78.70) | 72.85(67.79 to 77.91) | 0.90 (-6.14 to 7.93) |
| 12 months | 73.45 67.94 to 78.95) | 72.84(67.78 to 77.90) | 0.6 (-6.82 – 8.03) |
| Bodily pain |  |  |  |
| Baseline | 72.39 (68.11 to 76.66) | 71.09 (67.36 to 74.83) | 1.30 (-4.34 to 6.93) |
| 12 months | 72.93 (68.76 to 77.10) | 69.31 (65.64 to 72.97) | 3.62 (-1.89 to 9.13) |
| General health |  |  |  |
| Baseline | 66.17 (62.45 to 69.89) | 66.35 (62.87 to 69.84) | -0.18 (5.24 to 4.88 |
| 12 months | 70.42 (66.85 to 73.99) | 68.66 (65.57 to 71.75) | 1.76 (-2.92 to 6.45) |
| Vitality |  |  |  |
| Baseline | 65.39 (62.72 to 68.05) | 61.18(58.19 to 64.17) | **4.20 (0.23 to 8.18)*** |
| 12 months | 65.53 (62.45 to 68.61) | 60.49 (57.39 to 63.58) | **5.04 (0.71 to 9.37)*** |
| Social functioning |  |  |  |
| Baseline | 78.31 (74.05 to 82.58) | 74.47 (69.79 to 79.14) | 3.85 (-2.44 to 10.13) |
| 12 months | 80.27 (76.24 to 84.29) | 75.11 (70.66 to 79.56) | 5.15 (-0.80 to 11.11) |
| Role emotional |  |  |  |
| Baseline | 78.70 (73.73 to 83.67) | 76.14 (70.99 to 81.29) | 2.56 (-4.55 to 9.67) |
| 12 months | 80.52 (75.45 to 85.58) | 74.52 (69.43 to 79.62) | 5.99 (-1.14 to 13.13) |
| Mental health |  |  |  |
| Baseline | 73.61 (71.02 to 76.21) | 70.97 (67.39 to 74.54) | 2.65 (-1.74 to 7.04) |
| 12 months | 75.12 (72.16 to 78.08) | 71.53 (68.25 to 74.82) | 3.59 (-0.81 to 7.98) |
| Physical component score |  |  |  |
| Baseline | 49.22 (47.76 to 50.67) | 48.94 (47.40 to 50.49) | 0.27 (-1.83 to 2.38) |
| 12 months | 48.99 (47.51 to 50.47) | 49.06 (47.79 to 50.32) | -0.07 (-1.99 to 1.86) |
| Mental component score |  |  |  |
| Baseline | 50.22 (48.71 to 51.72) | 48.47 (46.47 to 50.46) | 1.75 (-0.73 to 4.23) |
| 12 months | 51.34 (49.81 to 52.86) | 48.39 (46.54 to 50.23) | **2.95 (0.57 to 5.33)*** |

* Significant at p < 0.05

^a^ Determined with linear mixed effect
